# Critical Transitions: A Mixed Methods Examination of Sleep from Inpatient Alcohol Rehabilitation Treatment to the Community

**DOI:** 10.1371/journal.pone.0161725

**Published:** 2016-08-29

**Authors:** Alyssa Todaro Brooks, Michael Krumlauf, Craig S. Fryer, Kenneth H. Beck, Li Yang, Vijay A. Ramchandani, Gwenyth R. Wallen

**Affiliations:** 1 National Institutes of Health Clinical Center, Bethesda, Maryland, United States of America; 2 National Institute on Alcohol Abuse and Alcoholism, Bethesda, Maryland, United States of America; 3 University of Maryland School of Public Health, Department of Behavioral and Community Health, College Park, Maryland, United States of America; University of Granada, SPAIN

## Abstract

**Aims:**

This prospective, repeated measures study utilized a convergent parallel mixed methods approach to assess sleep experiences among individuals who were alcohol-dependent undergoing inpatient detoxification and treatment at a clinical research facility across the transition periods associated with the rehabilitation process: the initial adjustment to becoming an inpatient and the transition from inpatient to outpatient status.

**Methods:**

This study included individual semi-structured interviews and quantitative measures relating to psychological distress, sleep quality, daytime sleepiness, and sleep-related beliefs and behavior (n = 33; 66.7% male). Interviews were conducted and questionnaires were administered within one week of participants’ scheduled discharge date and again four to six weeks post-discharge when they returned for a follow-up visit (or via phone).

**Results:**

Participants self-reported significant sleep disturbances at both study time points. Of those participants with valid data at both time points (n = 28), there were no significant changes in mean scores from pre- to post-discharge with the exception of self-efficacy for sleep (SE-S) being significantly higher post-discharge. Preliminary qualitative findings suggested differences between those with ongoing sleep disturbances, those whose sleep disturbances had resolved, and those with no sleep disturbances at either time point.

**Conclusions:**

This analysis highlights individual variation in sleep throughout the process of inpatient treatment and transition to outpatient aftercare in individuals with alcohol dependence. Collecting quantitative and qualitative data concurrently and combining emerging themes from qualitative data with quantitative analyses allowed for a more thorough examination of this relatively novel area of research and provided information that can be utilized to inform future behavioral sleep interventions.

## Introduction

Alcoholism, a chronic and progressive disease, is often accompanied by co-morbid conditions such as sleep disturbances [1–3]. Alcohol use can negatively affect sleep via increased nightmares, snoring, and other interruptions [4]. Sleep disturbances are common during phases of drinking and recovery [5], can persist for months or years during the process of recovery [4], and are especially common among individuals who are alcohol-dependent with co-morbid depression [6]. Reduced sleep is common during withdrawal after chronic abuse of alcohol [7], even after the “acute” withdrawal period [8]. Abstinence and “moderate” drinking significantly predict a reduction of insomnia symptoms in some patients, but symptoms may persist even after achieving abstinence [9]. Interestingly, in one study, *subjective* sleep measures were better predictors of future drinking (relapse) than objective measures (polysomnography) [10].

Insomnia is defined at the most basic level as difficulty falling or staying asleep and is relatively common in the U.S. [11]. Sleep disturbances are related to a multitude of health problems and can greatly reduce an individual’s quality of life [12–15]. Among treatment-seeking individuals who are alcohol-dependent, insomnia symptoms may increase psychosocial consequences related to alcohol [16]. Both pharmacologic and non-pharmacologic treatment options exist for individuals who are alcohol-dependent suffering with sleep disturbances ([17].

### Relationship between sleep disturbances and relapse to drinking

Sleep disturbances among individuals who are alcohol-dependent may be associated with increased risk of relapse to drinking following detoxification and rehabilitation [4, 18, 19]). Baseline sleep problems upon entering inpatient treatment have predicted subsequent relapse to drinking [18, 20]. Insomnia and sleep fragmentation after a period of abstinence may be related to relapse 14 months following abstinence [21]. For some individuals, re-initiation of drinking after achieving abstinence may be an attempt to self-medicate for disrupted sleep [22], and sleep-related behaviors such as the use of alcohol to help fall asleep have been associated with relapse 12 months after discharge from a residential addiction treatment program [23]. The longer-term consequences of sleep disturbances among individuals who are alcohol-dependent remain unclear.

### Transition periods during the alcohol rehabilitation process

For individuals who are alcohol-dependent seeking inpatient treatment, both the initial adjustment to inpatient treatment and the transition from inpatient to outpatient status are time periods accompanied by many changes. The environment and structure of an inpatient facility warrant certain adjustments on the part of the individual. Upon discharge from an inpatient facility, continuity of care with diverse programmatic support structures have been utilized to help individuals maintain healthy lifestyle changes and maximize the likelihood of sustained sobriety [24]). “Transition groups” have been used to engage individuals in the process of transitioning from inpatient facilities to discharge into outpatient care [25], but many of these programs are focused solely on preventing relapse. Beyond the intent to remain sober, access to transportation for continuing care appointments, success of inpatient treatment, and motivation levels are all factors which may influence sobriety [26]. Getting adequate, quality sleep is not only an important component of a healthy lifestyle but, as demonstrated by previous literature [4, 18, 19], might also play a role in preventing relapse. Thus, it may be beneficial for clinicians to assess sleep patterns and any changes in sleep throughout the rehabilitation process should be monitored.

### Potential value of mixed methods research / study design

Exploring individual perspectives and experiences could provide a better understanding of sleep throughout recovery [17]. In order to understand complex phenomena such as sleep disturbances and alcoholism, qualitative research that is naturalistic and subjective in nature *combined* with deductive quantitative techniques moves beyond traditional approaches and could potentially increase our knowledgebase [27]. Collecting both types of data concurrently and comparing emerging themes from qualitative data with quantitative analyses allows for a more thorough understanding of the complexity of sleep and the impact of any individual differences. This prospective, repeated measures study utilized a convergent parallel mixed methods approach to assess sleep experiences among individuals who were alcohol-dependent undergoing inpatient detoxification and treatment at a clinical research facility across the transition periods associated with the rehabilitation process: the initial adjustment to becoming an inpatient and the transition from inpatient to outpatient status.

## Methods

This study was approved by the NIH Addictions Institutional Review Board (IRB) at the National Institutes of Health (NIH; NCT # 02181659). All participants enrolled in this study were first admitted to a clinical research facility providing inpatient detoxification and treatment under a screening and assessment protocol, which enrolls adults over 18 years of age seeking treatment for alcohol dependence. All participants received continued physical evaluations, inpatient treatment of alcohol withdrawal, psychosocial management, and an educational treatment program. Participants were eligible to receive up to six or more weeks of inpatient treatment followed by 16 weeks of optional outpatient treatment. Participants were paid for the study portions they completed based on NIH guidelines [28]. All participants signed an informed consent document indicating their voluntary participation and understanding of study procedures and expectations.

### Inclusion and exclusion criteria

Participants were eligible for this study if they were 18 years of age or older, enrolled on the screening and treatment protocol (parent study), an inpatient for 21 days or more preceding discharge, not simultaneously enrolled onto a pharmacologic intervention study, able to understand the study and provide informed consent, and willing to return to the Clinical Center four to six weeks after being discharged from inpatient treatment for a follow-up visit *or* complete the follow-up study visit by phone.

### Study timeline

Specific measures already collected upon inpatient admission as part of the screening and assessment protocol were used to characterize individuals who participated in this study. Approximately one week prior to participants' scheduled discharge, a study team member approached participants to begin the first segment of data collection for the study. Interviews were conducted and questionnaires were administered within one week of participants’ scheduled discharge date and again four to six weeks post-discharge when they returned for a follow-up visit (or via phone).

### Qualitative measures

The qualitative component of this study was based on individual semi-structured interviews. The interview questions were reviewed and pilot-tested by clinicians and investigators with extensive experience working with alcohol-dependent individuals. A second interviewer (MK or GW) was present at all interviews and introduced to the participants with an explanation that he or she would observe, take notes, and probe additional questions based on the participant’s responses. This strategy was employed to decrease the potential bias of only having one interviewer. Questions were focused on sleep patterns prior to becoming an inpatient, during the inpatient stay, and in anticipation of becoming an outpatient and were designed to complement quantitative data. The first interview covered perceptions and descriptions of sleep in the clinic to the home environment, while the second interview focused on the participants’ support system as well as barriers and facilitators to both sleep and sobriety. All interviews were conducted by the first author (AB) for consistency, with the exception of one follow-up interview conducted by the second author (MK). The qualitative phase of data collection was always conducted first to ensure that participants’ responses would not be unduly influenced by their having read the sleep-related surveys prior to being interviewed.

### Quantitative measures

#### Baseline–psychological distress (collected during inpatient phase only)

Specific measures collected as part of the screening protocol were used to provide descriptive data on participants. The Comprehensive Psychopathological Rating Scale (CPRS) consists of 19 items that correspond to two CPRS-based subscales for affective and anxiety syndromes [29]: 1) the Montgomery Åsberg Depression Rating Scale (MADRS) [30] and 2) the Brief Scale for Anxiety (BSA) [31]. Overall scores range from 0 to 60 with higher scores indicative of more severe symptomatology.

The *Structured Clinical Interview for Diagnostics and Statistics Manual-IV (DSM-IV) (SCID-I)* is the standard interview to evaluate criteria for a psychiatric diagnosis, including that of alcohol dependence and disorders that are frequently co-morbid with alcohol dependence [32]. It is a structured interview consisting of 11 modules with between 35–292 items per module that takes about 120–180 minutes. Interviews are carried out by trained mental health professionals whose inter-rater reliability is continuously monitored. We assessed the number of both anxiety and mood disorders from the SCID-I. The *CIWA-AR*: *Clinical Institute Withdrawal Assessment-Alcohol Revised* is a validated tool is used to determine the severity of alcohol withdrawal based on symptoms and physical signs [33].

#### Sleep quality and daytime sleepiness (assessed approximately one week pre-discharge and 4–6 weeks post-discharge)

The Pittsburgh Sleep Quality Index (PSQI) is a 19-item, self-rated questionnaire used to measure sleep quality and disturbances over a one-month (30 days) time interval. A global summation score higher than five is indicative of poor sleep quality or “disturbed” sleep [34]. The PSQI has been extensively validated in populations with insomnia and other sleep disorders, with psychiatric patients, and in normal populations [35, 36]. Unlike all other assessments (which were administered at one week pre-discharge and/or 4–6 weeks post-discharge), the PSQI was administered at three different time points: baseline (day 2 of inpatient treatment), one week pre-discharge, and 4–6 weeks post-discharge. Internal reliability ranged from α = 0.576 to 0.840 at the pre- and post-discharge time points.

The Epworth Sleepiness Scale (ESS) is an eight-item self-administered questionnaire that provides a measure of an individual’s general level of excessive daytime sleepiness over a one week time period [37]. Individuals are asked to rate their usual chances of dozing off or falling asleep on a four-point scale in eight distinct situations or activities that most people engage in during their daily lives. Higher scores are indicative of higher levels of daytime sleepiness. A score higher than ten is indicative of “excessive” daytime sleepiness [38]. Internal reliability of the ESS was high in the current study (α = .704 to 0.794 at both time points).

#### Sleep-related beliefs and behaviors (assessed approximately one week pre-discharge and 4–6 weeks post-discharge)

The Dysfunctional Beliefs and Attitudes about Sleep Scale (brief version: DBAS-16) is a 16-item questionnaire that assesses sleep-related cognitions including faulty beliefs and appraisals, unrealistic expectations, and perceptual and attention bias [39]. Higher scores are indicative of stronger endorsement of dysfunctional beliefs. The internal reliability of the DBAS-16 was high in the current study (α = 0.831 to 0.888 at both time points). The Self-Efficacy for Sleep Scale (SE-S) includes nine items used to measure the level of confidence a person has in performing behaviors that might be helpful in initiating sleep, with higher scores indicative of greater confidence [40]. Internal reliability of the SE-S was high in the current study (α = 0.768 to 0.843 at both time points). The Sleep-Related Behaviours Questionnaire (SRBQ) assesses the use of safety behaviors that individuals may use to promote sleep and cope with tiredness [41]. Higher scores are indicative of higher frequency of engaging in safety behaviors in an effort to cope with sleeplessness or tiredness. The internal reliability of the SRBQ was high in the current study (α = 0.834 to 0.843 at both time points).

#### Alcohol-related measures–craving and relapse (collected 4–6 weeks post-discharge)

The Penn Alcohol Craving Scale (PACS) is a clinical tool for practitioners to measure alcohol craving. It is a five-item self-administered instrument that measures frequency, intensity, and duration of thoughts about drinking along with ability to resist drinking (possible range: 0–30) with demonstrably excellent internal consistency, predictive validity, construct validity, and discriminant validity [42]. The Timeline Follow-Back (TLFB) collects drinking information using personal historical events recounted over a fixed time period [43]. It is a standard assessment for measuring alcohol drinking patterns and quantification in treatment programs and was the primary measures of relapse for this study. If the TLFB was missing or invalid, we used a positive Breath Alcohol level or participants’ voluntarily self-reporting relapse during the second interview as indicators of relapse.

### Analyses—qualitative data

Each audio-recorded interview was transcribed and quality checked prior to analysis. A codebook was developed based on emergent themes related to transitions and changes in sleep over time from the interviews. A team of two coders independently reviewed a sub-set of transcripts. Discordant coding was discussed until consensus among the coding team was achieved. NVivo (version 10.0) was utilized for further qualitative analyses and to calculate inter-rater reliability percentages.

Once the iterative process of consensus building was complete, a representative from the clinical team and a mixed methods expert from the NIH Clinical Center validated the themes and codes presented herein. To ensure that the trustworthiness of qualitative data was preserved, three criteria assessing rigor were considered: creditability, auditability, and fittingness of the data [44].

### Analyses—quantitative data

Statistical analyses of quantitative results were conducted with the Statistical Package for Social Sciences (SPSS) software, version 22.0. All quantitative data were double-data entered, cross-checked, and reconciled where necessary. Sleep quality (PSQI) and relapse status (TLFB and other sources) were the main outcomes of interest. Paired t-tests and McNemar tests were used to compare pre- and post-discharge differences in these main outcomes. Mixed model repeated measure analyses were used to assess the sleep quality (PSQI) changes over three study time points. A p value < 0.05 was considered significant for all analyses. Missing data was assumed to be missing at random.

### Use of mixed methods–convergent parallel design

The format for the use of mixed methods was a *convergent parallel design*, wherein both quantitative and qualitative data were collected simultaneously and each method of examination was given equal priority [45]. Quantitative and qualitative results were merged during analysis and interpretation [46].

## Results

General demographics and clinical variables are presented in Table 1. On the second day of inpatient treatment, the average Pittsburgh Sleep Quality Index (PSQI) score was indicative of sleep disturbances (mean 12.0, s.d. 4.0). All individuals who were eligible to participate in the study agreed to participate and were enrolled. Three of the 33 participants reported being diagnosed with sleep apnea, one of whom also reported being diagnosed with nightmare disorder and restless legs syndrome. One additional participant reported substance/medication-induced sleep disorder. Of the 33 participants who completed the pre-discharge study visit, 28 (84.8%) returned for the post-discharge visit. The five participants who did not return were significantly more likely to be older (p < 0.05) and African-American (p < 0.05), but did not differ significantly from those who did return based on any other demographic, clinical, or sleep-related variables.

**Table 1:** Participant demographics and clinical variables (n = 33).*

		**n (%)**
**Gender**		
Male		22 (66.7)
Female		11 (33.3)
**Race/ethnicity**		
Black/African American		15 (45.4)
White		16 (48.4)
Other/multiracial		2 (6.0)
**Relapse** (post-discharge, n = 28)		
Relapse		7 (21.2)
No relapse		7 (21.2)
Missing		14 (42.4)
**Marital status**		
Single		22 (66.7)
Divorced		7 (21.2)
Married		3 (9.1)
Not provided		1 (3.0)
**PTSD** (not mutually exclusive categories)		
Current		4 (12.1)
Past		7 (21.2)
Lifetime		9 (27.3)
**Mood disorders** (SCID)+		18 (54.5)
**Anxiety disorders** (SCID)+		17 (51.5)
**Other substance use disorders** (SCID; excluding alcohol)		21 (63.6)
**Current cannabis use**		
Abuse		2 (6.1)
Dependence		2 (6.1)
**Current cocaine use**		
Dependence		1 (3.0)
	**Range**	**Mean (s.d.)**
**Age**	25–59 years	44.42 (10.43)
**TLFB–number of drinking days (out of 90)**	12–90 days	65.55 (26.88)
**TLFB–number of heavy drinking days (out of 90)**	11–90 days	62.70 (28.69)
**TLFB–average drinks per day** (Range: 4.2–33.0)	4.2–33.0 drinks	13.27 (5.95)
Baseline depression (CPRS)** (n = 32)	2–37	18.0 (7.7)
Baseline anxiety (CPRS)** (n = 32)	2–30	13.2 (6.6)
**CIWA**	0–20	6.60 (5.47)
**PACS** (post discharge, n = 28)	0–28	8.73 (8.71)

* If n ≠ 33 (data were missing), it is noted in the left column.

** “Baseline” denotes day 2 of inpatient treatment.

+Denotes proportion of participants with one or more mood/anxiety disorders.

**CPRS:** Comprehensive Psychopathological Rating Scale

**PACS:** Penn Alcohol Craving Scale

**CIWA:** Clinical Institute Withdrawal Assessment (maximum score over first four days of inpatient admission)

**TLFB:** Timeline Follow-Back

**SCID:** Structured Clinical Interview for DSM Disorders

Of the 28 participants who returned for the follow-up visit, 14 (50%) had data on relapse (from the Timeline Follow-Back-TLFB, breath alcohol content at the time of the follow-up visit, and/or having voluntarily admitted to drinking during their follow-up visit). Of the eight participants who had valid Timeline Follow-Back (TLFB) data at follow-up (4–6 weeks post-discharge), only one had relapsed within the 4–6 week post-discharge time frame, reporting drinking four of the 34 days with an average of 7.13 drinks per drinking day. The nine participants who did not mention drinking during their follow-up interview were treated as “missing” since verification of sobriety was not possible (denoted in Table 1).

### Qualitative results

The goal of achieving an inter-rater agreement of over 80% was met. Qualitative themes related to transitions and changes in sleep over time are presented, along the frequency of their endorsement at each time point, in S2 Table. It is important to note is that some themes did not “emerge” on their own but were actually prompted by the interviewer.

A majority of participants (72.7%) discussed some type of fear or uncertainty surrounding either the adjustment to becoming an inpatient and/or returning back home during the pre-discharge interview. Similarly, a large majority of participants (82.1%) discussed the level of difficulty or ease of their transition back home during their follow-up interviews. While only 18.2% of participants discussed a healthy lifestyle in the context of their recovery process during their inpatient admission, almost half of those who returned for the follow-up interview endorsed healthy lifestyle changes since returning home. Most participants (60.6%) identified *anticipated* barriers and facilitators to sobriety before leaving the inpatient facility, but fewer participants (33.3%) discussed the *actual* barriers and facilitators they had experienced during the follow-up interviews. Participants discussed sleep-related behaviors (i.e. bedtime routines and/or strategies for falling asleep or staying asleep) at both time points (81.8% at pre-discharge and 78.6% at post-discharge). A considerable number of participants 39.4% discussed “racing thoughts” while reflecting on their sleep or drinking patterns during the first interview.

### Quantitative results

**Fig 1:**
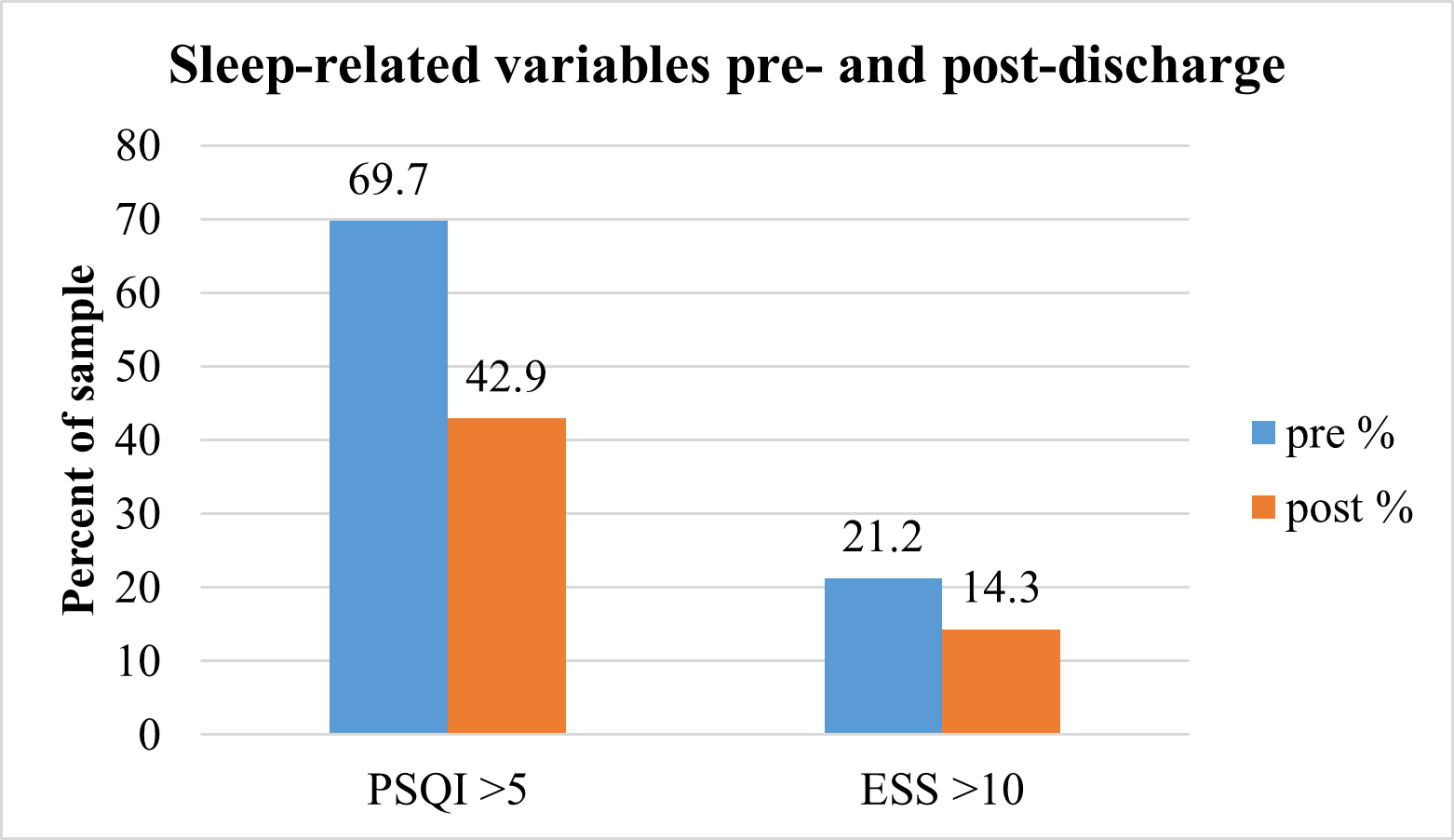
Sleep-related variables pre- and post-discharge. * *McNemar test performed only in the case of valid data at both time points (PSQI: n = 26; ESS: n = 28). Five (5) participants were lost to follow-up. p < 0.05 (change in distribution of PSQI scores).

We examined differences in sub-scales of the PSQI by time point in Table 2. McNemar tests were used to assess differences in the distribution of “sleep disturbances vs. no sleep disturbances” (PSQI) and “excessive daytime sleepiness vs. no excessive daytime sleepiness” (Epworth Sleepiness Scale: ESS). Among those with valid data at both time points (n = 26), the proportion of those with sleep disturbances as measured by the PSQI differed significantly from pre- to post-discharge (p < 0.05). There were no significant differences in the proportion of those with excessive daytime sleepiness at pre- and post-discharge as measured by the ESS (n = 28, Fig 1).

**Table 2:** Pittsburgh Sleep Quality Index (PSQI) sub-scales.

	Pre-discharge, mean (s.d.)	Post-discharge, mean (s.d.)
	n = 33*	n = 28**
**Global score**	7.50 (3.53)	6.35 (4.61)
**Sleep quality**	0.94 (0.72)	0.82 (0.86)
**Sleep latency**	1.61 (1.03)	1.39 (0.92)
**Sleep duration**	1.21 (1.08)	0.71 (0.98)
**Sleep efficiency**	0.73 (1.10)	0.61 (0.88)
**Sleep disturbance**	1.50 (0.67)	1.33 (0.62)
**Sleep medication**	0.61 (1.20)	0.81 (1.27)
**Daytime sleep dysfunction**	0.85 (0.62)	0.59 (0.69)

PSQI raw global scale range: 0–21; sub-score scale range: 0–3

*Sleep disturbance & global score pre-discharge; n = 32

**Sleep disturbance, sleep medication, daytime sleep dysfunction, & global score post-discharge, n = 27

The mean PSQI scores approximately one week pre-discharge and 4–6 weeks post-discharge remained above the cut-off for “disturbed sleep” (7.62 ± 3.70 and 6.35 ± 4.61, respectively; Fig 1). Of those participants with valid data at both time points (n = 26 for PSQI and n = 28 for all other sleep-related variables), there were no significant changes in mean scores on sleep-related variables from pre- to post-discharge with the exception of self-efficacy for sleep (SE-S) being significantly higher post-discharge (29.25 ± 6.70 versus 31.29 ± 7.62, p = 0.048). No differences were found between males and females when considering the change in PSQI scores from pre- to post-discharge. Interestingly, non-White participants were more likely to experience improvements in PSQI scores from pre- to post-discharge (p = 0.008).

In addition to the McNemar test, a repeated measures linear mixed model using 88 time points from all 33 cases found that the estimated marginal mean for baseline sleep disturbances as measured by the PSQI on day 2 of inpatient treatment (12.29, s.e. 0.73) was significantly higher than *one week* pre- (7.47, s.e. 0.71) and 4–6 weeks post-discharge (6.27, s.e. 0.77) PSQI scores (p < .001 for both comparisons). No significant difference was found between *one week* pre- and 4–6 weeks post-discharge.

There were no *statistically* significant differences in demographic or sleep-related variables between those who relapsed, those who were sober, and those whose relapse data were missing, likely due to the small sample size. Additionally, there were no differences in baseline average number of drinks per day in the 90 days prior to admission between the same groups (p-value from Kruskal-Wallis test = 0.48). However, demographic and clinical variables are presented by relapse status (if known) and “trajectory” of sleep quality based on PSQI scores pre- and post-discharge in Table 3. In the table, “ongoing sleep disturbances” refers to individuals whose PSQI scores were above the cut-off (> 5) for sleep disturbances at both time points. “Sleep disturbances resolved” refers to individuals whose pre-discharge PSQI score was above the cut-off for sleep disturbances but their post-discharge PSQI score was below the cut-off. Finally, “no sleep disturbances at either time point” refers to individuals whose PSQI scores were below the cut-off for sleep disturbances at both time points. Only one participant went from having no sleep disturbances pre-discharge to *developing* sleep disturbances post-discharge based on PSQI scores, although some participants outlined negative changes in sleep from pre- to post-discharge qualitatively. Those who relapsed had higher craving scores (Penn Alcohol Craving Scale—PACS) and those with no sleep disturbances at either time point had the lowest craving scores of any group, but the differences were not statistically significant. All of the participants whose sleep disturbances resolved were non-White.

**Table 3:** Participant demographics and clinical variables by relapse status and sleep quality.

**Relapse (n = 7)**		**No relapse (n = 7)**	
	**n (%)**		**n (%)**
Male	5 (71.4)	Male	4 (57.1)
Non-white	2 (28.6)	Non-white	4 (57.1)
	**Mean (s.d.)**		**Mean (s.d.)**
Age	43.29 (14.33)	Age	47.29 (9.76)
PACS	18.14 (7.90)*	PACS	4.29 (3.77)*

**Ongoing sleep disturbances (n = 11)**		**Sleep disturbances resolved (n = 8)**		**No sleep disturbances at either time point (n = 6)**	
	**n (%)**		**n (%)**		**n (%)**
Male	6 (54.5)	Male	6 (75.0)	Male	5 (83.3)
Non-white	1 (9.1)	Non-white	8 (100.0)	Non-white	1 (16.7)
	**Mean (s.d.)**				**Mean (s.d.)**
Age	44.73 (11.56)	Age	46.13 (10.55)	Age	45.00 (8.92)
PACS	11.59 (10.28)	PACS	6.75 (6.07)	PACS	3.17 (4.02)

*PACS (Penn Alcohol Craving Scale) was significantly higher among those who relapsed (p = .001).

Preliminary qualitative findings suggested differences between those with ongoing sleep disturbances (n = 11), those whose sleep disturbances had resolved (n = 8), and those with no sleep disturbances at either time point (n = 6). Only one of the eight individuals whose sleep disturbances resolved and two of the 11 individuals with ongoing sleep disturbances mentioned healthy lifestyles during their pre-discharge interviews. Only one of the eight individuals whose sleep disturbances had resolved mentioned “racing thoughts” during their pre-discharge interview. Lastly, to complement the quantitative results and summarize the overarching emergent themes from the interviews, we present key qualitative findings by theme, specifically those related to the transition from inpatient to outpatient, in Table 4.

**Table 4:** Summary of key qualitative findings by time point and theme.

**Pre-discharge qualitative themes**	**Prevalent findings**	**Sample quotes**
Fear / uncertainty related to transition to becoming an inpatient or returning home	• Initial adjustment period upon arriving to inpatient facility (new environment, new “rules”—for some, this was while undergoing medically-assisted detoxification and treatment for withdrawal) • This was often followed by an “adjustment” period and developing a level of comfort with the inpatient rehabilitation program routine • Anxiety, excitement, or a mixture of both feelings regarding the transition back home • Particularly among those who had not attempted sobriety before, some degree of uncertainty surrounding not knowing “triggers” to relapse, whereas those who had been sober before often focused on what they would do differently • Regardless of prior experiences with rehabilitation, participants placed a lot of emphasis on maintaining sobriety / managing stress as primary determinants of their success post-discharge	• “It took me a couple of days…to get adjusted…I observe things when I’m around new things or people… just to see how comfortable I can get.” -*34 year old African American female*, *pre-discharge* • “I would be disillusioned to tell you I got this thing figured out, or–anything like that…but…I can’t live here forever…” *-45 year old White male*, *pre-discharge*
Healthy lifestyle (structure, health behaviors, health information)	• Appreciation of the structure associated with the inpatient facility (regular meal times, normalizing sleep schedules, making time for physical activity) • Those with concerns about their physical health appreciated the clinical / diagnostic tests and receiving information on their health	• *“I have more energy… I focus my energy on more positive things…now*, *because drinking is eliminated*, *I’m using that…extra energy for good things*. *I go to the gym*, *I play basketball out back*, *I got my bike here on campus*, *I go bike riding…”* -37 year old White male, pre-discharge • *“Definitely now at this point*, *I…feel that I am on the right path*. *And I just have a new outlook on life*. *So*, *that’s where I am now…I’m a different person that I walked in here…30 –uh*, *28 days ago*.*”* -53 year old African American female, pre-discharge
Sleep-related behavior (relaxation strategies and sleep hygiene techniques)	• Initiation of bed-time routines or other sleep-related behaviors during the inpatient stay (use of relaxation techniques, herbal / pharmacological remedies, attempting to implement a regular sleep schedule, etc) • Anticipating the continuation of these behaviors post-discharge	• *“I go to sleep with…a little bit more contentment…I’ll put it that way…when I get up in the morning*, *I used to dance every morning in my bedroom*. *And I haven’t done that in the last three to four years*. *So*, *this morning I found myself dancing before I got dressed…that was cool*.*”* -53 year old African American female, pre-discharge • *“I’m back into meditation now…and I’ve also got chamomile tea*. *But I’m looking forward to sleep now*. *And I’ve got tools to cope with now*.*”* -53 year old African American female, pre-discharge
Mind or thoughts racing	• Racing thoughts / inability to stop thinking, either when trying to go to sleep or as a precursor to drinking	• *“I couldn’t really go to sleep ‘cause my thinking was just…here come the thoughts again…a racket going on in my mind*.*”* -55 year old African American male, pre-discharge • *“Sleep’s terrible*, *you know*, *‘cause your mind’s racing–with all the things that are really going on…”* -47 year old African American male, pre-discharge
**Post-discharge themes**	**Prevalent findings**	
Transition back home	• Feeling overwhelmed with the stress of “normal” life—including job interviews, family stressors, and other aspects of their lives they had been away from for at least 3 weeks • In some cases, these stressors led to relapse • Finding a job or finding purpose / meaning in other activities (e.g. re-connecting with family or volunteering in the community) were motivators to stay sober • Those who had not relapsed at the time of the second interview were more likely to perceive other aspects of the transition back home more positively • Participants indicated there were both “ups” and “downs” (positives and negatives) related to this transition	• *“A lot of times*, *I think about drinking and things like that*, *and just–I knew it was wrong*, *but I still would just follow my impulses*. *Now*, *like–I still get cravings every now and then*, *but every time they go in my head I think about all the bad stuff that happened*, *and they’ll go away*.*”* -27 year old African American male • *“[The] transition was a little rough*. *I got out…I went to stay with my cousin for a little bit…and then started drinking again*, *um…so that fell through…I have had one slip-up since I’ve been there…besides that…[I’m] just really trying to focus on sobriety*.*”* -27 year old White male, post-discharge
Lifestyle changes (health behaviors)	• Healthy lifestyle changes mentioned during the second interview included being sober, having non-alcohol methods of coping with stressful situations, losing weight, increased physical activity, drinking less coffee, re-organizing living spaces or finances	• *“I’m not waking up looking for the vodka bottle*. *That’s…the blunt way to put it…I’ve filled my life with other things*, *and yes*, *you can still have fun without drinking*.*”* -53 year old African-American female, post-discharge • *“I’m eating much better…much healthier*. *And I’m still exercising almost every day*, *whether it’s–I mean*, *just taking a walk–and I think those two things are kind of important*, *um…at least*, *an overall balance*, *and I think–it definitely helps me*.*”* -50 year old White male, post-discharge
Sleep-related behavior (relaxation strategies and sleep hygiene techniques)	• Some participants continued pre-bedtime rituals they initiated as inpatients, including drinking chamomile tea, meditation, calming music, guided imagery, reading, watching TV, and other methods of relaxation	• *“I still do try to meditate before I go to bed… and sometimes that… just calms me down*, *sometimes it doesn’t*. *It really depends on*, *I think*, *what I went through [during] the day*.*” -*49 year old White female, post-discharge • “*And I actually went and bought one of the little tapes that they recommended…it’s just got a bunch of*, *like*, *sea sounds in it*. *Bird chirps…”* -47 year old African American male, post-discharge
Overarching changes in sleep (since leaving inpatient facility)	• Drunk dreams, dreaming more frequently (sometimes attributed to a change in medication) • Improved sleep (less interruptions, comfortable environment) • Many who had maintained sobriety felt that sleep was more regular / routine • Stress of the transition back home and everyday life potentially led to increased sleep interruptions	• *“I will drink and then pass out–it’s not sleeping*, *it’s being passed out from alcohol being infused into my whole system*. *And it’s not a deep sleep*, *it’s more of a knocked out sleep*. *And then*, *after a few hours–I mean*, *this is not eight*, *nine hours–after a few hours I wake up…I can also tell in my face…when I’m drinking and I don’t sleep well*, *which is–always happens…I notice bags under my eyes*.*”* -57 year old White female, post-discharge

## Discussion

This analysis highlights individual variation in sleep throughout the process of inpatient treatment and transition to outpatient aftercare in individuals with alcohol dependence. Collecting quantitative and qualitative data concurrently and combining emerging themes from qualitative data with quantitative analyses through triangulation (simultaneously) allowed for a more thorough examination of this relatively novel area of research and provided information that can be utilized to inform future behavioral sleep interventions. As previously discussed, the rehabilitation process represents a time of transformation. Inpatient facilities may represent an environment conducive to initiating lifestyle changes, including those which could improve sleep.

As with previous studies [1, 47], the individuals in this study had a wide range of co-morbid conditions. Our findings support the co-occurrence of alcohol use and sleep disturbances [5, 48], particularly in the early stage of recovery [6, 49]. Individuals undergoing inpatient alcohol treatment are in a new environment, away from their homes and communities, and may therefore become accustomed to a “schedule” for eating, sleeping, and recreation. The first few days of the inpatient stay may not be the best time to introduce an intervention, as participants may be focused on adjusting to their surroundings. As evidenced by our results, the period of transition from inpatient to outpatient treatment represents another transition period of uncertainty and change. Sustaining healthy behaviors including sleep which may have been initiated during inpatient treatment could help to maintain sobriety, a healthy lifestyle, and overall health-related quality of life.

Similar to our previous work establishing the prevalence of sleep disturbances throughout the inpatient stay, there was low variability in daytime sleepiness levels (ESS) and average scores on the measure were not indicative of “excessive” daytime sleepiness. Measures of sleep-related safety behaviors (SRBQ) and dysfunctional beliefs about sleep (DBAS-16) remained relatively stable at both time points, despite participants discussing many changes occurring throughout the transition from inpatient to outpatient during their interviews. Self-efficacy for sleep (SE-S) improved significantly with inpatient standard of care which did not include specific interventions focused on sleep hygiene. However, study participants discussed bedtime routines and strategies for sleep they initiated as inpatients. In the inpatient program, it is possible that staff members could have provided suggestions for non-pharmacologic interventions. Additionally, there is a “schedule” for the unit when activities end each night and begin the next morning–and patients typically adjust accordingly. Despite these potential confounding factors, self-efficacy will be explored as an important variable in sleep behavior change in a larger sample, given its sensitivity in this small sample.

The demographic and clinical variables presented in Table 3 did not differ significantly by relapse status or sleep quality, but one trend emerged that could have clinical implications and warrants further investigation: those who self-reported no sleep disturbances at either time point had the lowest craving scores. Craving may be important to examine at various points throughout the recovery process, especially when considering the possibility of relapse. Furthermore, measures of sleep quality (PSQI) and daytime sleepiness (ESS) both trended toward reduction in the prevalence from pre- to post-discharge. Additionally, several interesting qualitative differences emerged by sleep quality status: 1) those who mentioned healthy lifestyles pre-discharge were less likely to self-report *no* sleep disturbances at either time point and 2) those whose sleep disturbances resolved by the post-discharge time point were less likely to endorse “racing thoughts” while inpatients. These findings offer preliminary support to the idea that “stabilizing” sleep during the inpatient phase may be especially important.

### Strengths and limitations

The mixed methods nature of this study allowed for the collection of rich and diverse information. In particular, the wealth of qualitative data accrued and the rigorous process through which they were analyzed was useful in describing the complex phenomenon of sleep throughout recovery. However, this study is not without limitations. Given a small sample size and subsequently low power levels, the quantitative analyses were strictly exploratory in nature and were meant to complement qualitative findings. The sample was non-representative; findings should not be generalized to all individuals who are alcohol-dependent, seeking treatment, and willing to return for a follow-up visit. Finally, at discharge, all but three participants were prescribed at least one medication that could have potentially altered their sleep. It is unclear how this use of sleep medications affected the nature of the qualitative narratives provided by the participants.

### Future directions

Future efforts should include following individuals for a longer period of time post-discharge when possible to capture participants’ experiences longitudinally and explore whether lasting changes to sleep parameters occur. The quotes reflected in this paper coalesce around the overarching theme of structure; the appreciation of re-developing routines during inpatient treatment may be conducive to changing sleep habits through a tailored behavioral sleep intervention. At a minimum, sleep should be monitored in alcohol treatment programs in order to understand it both as a potential relapse trigger and an important component of a healthy lifestyle post-discharge. The most effective mechanism for assessing sleep in this particular population (i.e. objectively, subjectively, or a combination of both) still needs to be explored.

### Conclusion

This research confirms a high prevalence of sleep disturbances in a sample of treatment seeking individuals who are alcohol-dependent throughout various stages of recovery that had not yet been explored in detail using a mixed methods approach. Patient-reported outcomes and in-depth interviews provided a clearer picture of individual experiences throughout recovery and the complexity of alcoholism and associated co-morbid conditions, compared to either data source individually. Capturing the essence of *transition* periods throughout the process of recovery highlights the important role they may play in sleep quality and eventually relapse. Of particular importance may be learning how to capitalize on positive lifestyle changes post-discharge from inpatient rehabilitation facilities that emerged qualitatively in this study, particularly those changes introduced during the inpatient phase of recovery and sustained. This study fills a gap in the literature by characterizing sleep throughout the rehabilitation process and ongoing maintenance of abstinence (or relapse).

## Supporting Information

S1 Table Interview prompts. Description of interviewer prompts for both the pre-and post-discharge interviews.
(DOCX)

S2 Table Qualitative themes specific to transitions and sleep changes over time. Description of themes relevant to transition from pre- to post-discharge and corresponding frequency of endorsements.
(DOCX)
