# Supplementary material for: Critical Transitions: A Mixed Methods Examination of Sleep from Inpatient Alcohol Rehabilitation Treatment to the Community
Source: PLoS One. 2016 Aug 29;11(8):e0161725. doi: 10.1371/journal.pone.0161725 (PMC5003361; doi:10.1371/journal.pone.0161725)
Supplement: S1 Table — Description of interviewer prompts for both the pre-and post-discharge interviews. (DOCX) [file pone.0161725.s001.docx]

**S1 Table. Interview Prompts.**

| **Interview #1** (conducted within a week of scheduled discharge) |
| --- |
| - Thank you for participating in this study. My name is ____________ and I'll be facilitating the interview today. ___________ is with me to observe and take some notes. We are audio-recording this session because we don't want to miss any of your comments. - We're conducting these interviews because we're trying to understand more about your recovery, transition into the community, and sleep patterns. Some questions are very general and others are more specific to your transition. I want you to talk out what you are thinking more than what you might normally be used to - so basically, to "think out loud." I do have a set of questions written out, but mainly, I just want to hear what you have to say. There are no “right” or “wrong” answers. If you are uncomfortable with any question, we can skip it. However, the more information you are able to provide me with, the more I'll be able to understand your experience and how each person's recovery process differs. You can choose to end the interview at any time and this will not affect your treatment here at NIH.   - What should I know about you as a person?   - Describe the process of how you adjusted to being an inpatient in the Clinical Center.   - Talk me through what your experience has been throughout the process of rehabilitation here at NIH.   - Think about the first few days after you leave here and go home. Walk me through what you think it will be like.   - Describe in as much detail as you can any expectations you have about transitioning back into your home environment. (Probe: What barriers or facilitators to recovery do you expect?)   - How did you sleep last night? Describe in as much detail as you can.   - Talk me through what your experience has been with sleep throughout the process of rehabilitation here at NIH.   - Describe how it has been to adjust to sleeping in this hospital. What about it is the same or different from your home environment? (Probe: positives and negatives of both environments...)   - Describe what you think your sleep will be like when you leave the NIH.   - Describe what you will do if you have trouble sleeping when you get home.   - When you feel like you have had a really "good night's sleep," what does that generally mean to you? Describe this in as much detail as you can. (Probe: how many hours, how you felt in the morning, ease of falling asleep...)   - How do you think alcohol affects sleep?   - Is there anything you'd like to add that we haven't discussed already? |
| **Interview #2** (conducted approximately one month post-discharge) |
| - Thank you for participating in this study. My name is ____________ and I'll be facilitating the interview today. ___________ is with me to observe and take some notes. We are audio-recording this session because we don't want to miss any of your comments. - We're conducting these interviews because we're trying to understand more about your recovery, transition into the community, and sleep patterns. Some questions are very general and others are more specific to your transition. I want you to talk out what you are thinking more than what you might normally be used to - so basically, to "think out loud." I do have a set of questions written out, but mainly, I just want to hear what you have to say. There are no “right” or “wrong” answers. If you are uncomfortable with any question, we can skip it. However, the more information you are able to provide me with, the more I'll be able to understand your experience and how each person's recovery process differs. You can choose to end the interview at any time and this will not affect your treatment here at NIH.   - It's been about a month since you left the Clinical Center. Talk me through what the transition has been like as you returned to your home environment. ( Probe: Different environment, support system.../ Describe any barriers or facilitators to recovery you have experienced.)   - How did you sleep last night? Describe in as much detail as you can.   - Compared to what it was like in the hospital, what has your sleep been like since leaving the NIH? (Probe: More/less sleep, more/less tired, dreams...)   - Describe what your process has been if you have experienced any trouble sleeping. (Probe: television, reading, medication, alcohol...)   - When you feel like you have had a really "good night's sleep," what does that generally mean to you? Describe this in as much detail as you can. (Probe: how many hours, how you felt in the morning, ease of falling asleep...)   - How do you think alcohol affects sleep?   - Would you be open to an intervention to help you sleep? What would an ideal sleep intervention look like to you? (Probe: Inpatient or outpatient, group-based or one-on-one, setting, timing...)   - Is there anything you'd like to add that we haven't discussed already? |
