# Supplementary material for: Critical Transitions: A Mixed Methods Examination of Sleep from Inpatient Alcohol Rehabilitation Treatment to the Community
Source: PLoS One. 2016 Aug 29;11(8):e0161725. doi: 10.1371/journal.pone.0161725 (PMC5003361; doi:10.1371/journal.pone.0161725)
Supplement: S2 Table — Description of themes relevant to transition from pre- to post-discharge and corresponding frequency of endorsements. (DOCX) [file pone.0161725.s002.docx]

**S2 Table. Qualitative themes specific to transitions and sleep changes over time.**

| **Pre-discharge** | | **Post-discharge** | |
| --- | --- | --- | --- |
| **Themes** | **Frequency of endorsement; n(%), (n=33)** | **Themes** | **Frequency of endorsement; n(%), (n=28)** |
| Fear / uncertainty related to transition to becoming an inpatient or returning home | 24 (72.7) | Transition back home | 23 (82.1) |
| Healthy lifestyle | 6 (18.2) | Lifestyle changes | 12 (42.9) |
| Anticipated barriers / facilitators to sobriety (family, social, other) | 20 (60.6) | Perceived barriers / facilitators to sobriety (family, social, other) | 8 (28.6) |
| Sleep-related behavior | 27 (81.8) | Sleep-related behavior | 22 (78.6) |
| Mind or thoughts racing | 13 (39.3) | Changes in sleep (since leaving inpatient facility) | 24 (85.7) |
